# Limited cross-variant immunity from SARS-CoV-2 Omicron without vaccination

**DOI:** 10.1038/s41586-022-04865-0

**Published:** 2022-05-18

**Authors:** Rahul K. Suryawanshi, Irene P. Chen, Tongcui Ma, Abdullah M. Syed, Noah Brazer, Prachi Saldhi, Camille R. Simoneau, Alison Ciling, Mir M. Khalid, Bharath Sreekumar, Pei-Yi Chen, G. Renuka Kumar, Mauricio Montano, Ronne Gascon, Chia-Lin Tsou, Miguel A. Garcia-Knight, Alicia Sotomayor-Gonzalez, Venice Servellita, Amelia Gliwa, Jenny Nguyen, Ines Silva, Bilal Milbes, Noah Kojima, Victoria Hess, Maria Shacreaw, Lauren Lopez, Matthew Brobeck, Fred Turner, Frank W. Soveg, Ashley F. George, Xiaohui Fang, Mazharul Maishan, Michael Matthay, Mary Kate Morris, Debra Wadford, Carl Hanson, Warner C. Greene, Raul Andino, Lee Spraggon, Nadia R. Roan, Charles Y. Chiu, Jennifer A. Doudna, Melanie Ott

**Affiliations:** 1 Gladstone Institutes, San Francisco, CA USA; 2 Biomedical Sciences Graduate Program, University of California, San Francisco, San Francisco, CA USA; 3 Department of Medicine, University of California, San Francisco, San Francisco, CA USA; 4 Quantitative Biosciences Institute COVID-19 Research Group, University of California, San Francisco, San Francisco, CA USA; 5 UCSF-Abbott Viral Diagnostics and Discovery Center, San Francisco, CA USA; 6 Innovative Genomics Institute, University of California, Berkeley, Berkeley, CA USA; 7 Department of Laboratory Medicine, University of California, San Francisco, San Francisco, CA USA; 8 Michael Hulton Center for HIV Cure Research at Gladstone, San Francisco, CA USA; 9 Department of Microbiology and Immunology, University of California, San Francisco, San Francisco, CA USA; 10 Curative Inc., San Dimas, CA USA; 11 Department of Urology, University of California, San Francisco, San Francisco, CA USA; 12 Department of Medicine, Cardiovascular Research Institute, University of California, San Francisco, San Francisco, CA USA; 13 Department of Anesthesia, Cardiovascular Research Institute, University of California, San Francisco, San Francisco, CA USA; 14 California Department of Public Health, Richmond, CA USA; 15 Chan Zuckerberg Biohub, San Francisco, CA USA; 16 Department of Molecular and Cell Biology, University of California, Berkeley, Berkeley, CA USA; 17 Molecular Biophysics and Integrated Bioimaging Division, Lawrence Berkeley National Laboratory, Berkeley, CA USA; 18 Howard Hughes Medical Institute, University of California, Berkeley, Berkeley, CA USA; 19 Department of Chemistry, University of California, Berkeley, Berkeley, CA USA; 20 California Institute for Quantitative Biosciences, University of California, Berkeley, Berkeley, CA USA; 21 Department of Medicine, University of California, Los Angeles, Los Angeles, CA USA

**Keywords:** Viral infection, SARS-CoV-2

## Abstract

SARS-CoV-2 Delta and Omicron are globally relevant variants of concern. Although individuals infected with Delta are at risk of developing severe lung disease, infection with Omicron often causes milder symptoms, especially in vaccinated individuals^1,2^. The question arises of whether widespread Omicron infections could lead to future cross-variant protection, accelerating the end of the pandemic. Here we show that without vaccination, infection with Omicron induces a limited humoral immune response in mice and humans. Sera from mice overexpressing the human ACE2 receptor and infected with Omicron neutralize only Omicron, but not other variants of concern, whereas broader cross-variant neutralization was observed after WA1 and Delta infections. Unlike WA1 and Delta, Omicron replicates to low levels in the lungs and brains of infected animals, leading to mild disease with reduced expression of pro-inflammatory cytokines and diminished activation of lung-resident T cells. Sera from individuals who were unvaccinated and infected with Omicron show the same limited neutralization of only Omicron itself. By contrast, Omicron breakthrough infections induce overall higher neutralization titres against all variants of concern. Our results demonstrate that Omicron infection enhances pre-existing immunity elicited by vaccines but, on its own, may not confer broad protection against non-Omicron variants in unvaccinated individuals.

## Abstract (web summary)

Infection with Omicron after vaccination produces cross-neutralizing antibodies to other variants of concern, whereas this induces a limited response to non-Omicron variants in unvaccinated individuals.

## Main

Since the beginning of the COVID-19 pandemic, multiple waves of infection have occurred from SARS-CoV-2 variants of concern (VOCs) that continue to arise and out-compete preceding variants. VOCs with worldwide relevance are Delta (B.1.617.2) and most recently Omicron (BA.1), whereas Alpha (B.1.1.7), Beta (B.1.351) and Gamma (P.1) variants spread more locally. Compared to the ancestral isolate (WA1 or B.1), Omicron is characterized by a large number of unique mutations in the spike protein as well as in other structural proteins, select non-structural proteins and accessory open reading frames. Omicron bears over 50 mutations across its genome, including approximately 37 mutations (28 being unique and nine overlapping with other variants) in the spike glycoprotein, which may contribute to its antigenic differences^3–9^.

The constellation of mutations in the Omicron spike protein has been associated with increased transmission^10^, decreased spike cleavage^11^ and decreased cell-to-cell fusion^11,12^. Omicron spike mutations limit efficacies of neutralizing antibodies generated by previous infections, vaccines and treatment with monoclonal antibodies^3–9,13^. Indeed, the risk of breakthrough infections and re-infections is increased with Omicron^13–15^. However, disease severity is lower with Omicron than with Delta^1,2,13^, and previous infection or vaccination reduces the risk of hospitalization with Omicron^16,17^. Pressing questions are how effective Omicron-induced immunity is and whether it is cross-protective against other variants.

## Omicron causes less severe infection

**Fig. 1:**
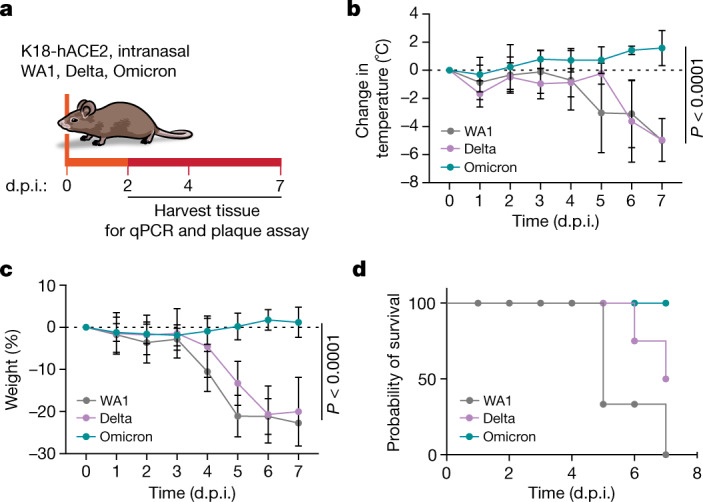
Robust infection of K18-hACE2 mice with the Delta and ancestral variants, but not with the Omicron variant. **a**, Schematic of the experiment. Fifteen mice per group were intranasally infected with 10^4^ p.f.u. of the indicated variant. Body temperature and weight were monitored daily. At 2, 4 and 7 days post-infection (d.p.i.), the upper respiratory tract and lungs were harvested and processed for downstream analysis. *n* = 5 per group. **b**, Changes in body temperature of mice infected with WA1, Delta and Omicron. Data are shown as the average ± s.d. and were analysed by two-way analysis of variance (ANOVA) and adjusted for multiple testing using the Bonferroni test. **c**, Severe weight loss of WA1-infected and Delta-infected mice. Data are shown as the average ± s.d. and were analysed by two-way ANOVA and adjusted for multiple testing using the Bonferroni test. The horizontal dashed lines in **b**,**c** indicate the baseline body temperature (**b**) and weight (**c**) of mice. **d**, Probability of survival of variant-infected mice. *n* = 10. Source Data

**Extended Data Fig. 1:**
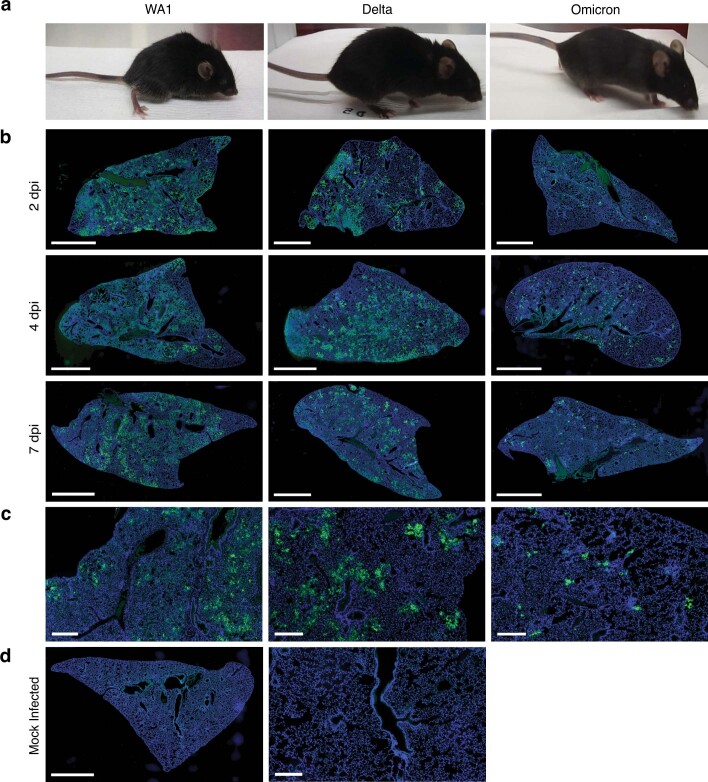
Physical conditions of the infection mice at 5 dpi. **a**, Representative images of WA1-, Delta-, and Omicron-infected mice 5 dpi. WA1-infected mice were lethargic and had a hunched posture, ungroomed coat, and squinted eyes. Delta-infected mice are mildly lethargic. Omicron-infected mice appeared normal. **b**, Representative images of lungs from mice infected with WA1, Delta, or Omicron at 2 dpi (n = 5), 4 dpi (n = 5), and 7 dpi (WA1 infection group n = 2, Delta n = 2 and Omicron n = 5). SARS-CoV-2 nucleocapsid is stained in green and nucleus is stained in blue. Scale bar, 2 mm. **c**, Representative images of tissue sections from lung tissue infected with WA1, Delta, or Omicron collected at 7 dpi (WA1 infection group n = 2, Delta n = 2 and Omicron n = 5). SARS-CoV-2 nucleocapsid is stained in green and nucleus is stained in blue. Scale bar, 300 μm. **d**, Representative images of mock infected lungs. SARS-CoV-2 nucleocapsid is stained in green and nucleus is stained in blue. Scale bar, 2 mm (left panel) and 300 μm (right panel), n = 5 mice.

To answer these questions, we studied WA1, Delta and Omicron infections in mice. Because WA1 and Delta variants cannot infect regular laboratory mice^18^, we used transgenic mice overexpressing human ACE2 (K18-hACE2)^19^. We intranasally infected (10^4^ plague-forming units (p.f.u.)) these mice with the three viral isolates, and over 7 days monitored their body temperature and weight, which serve as indicators of disease progression (Fig. 1a). Although Delta-infected and WA1-infected mice showed progressive hypothermia and severe weight loss during this time, Omicron-infected mice exhibited very mild symptoms with only a small increase in body temperature and no weight loss (Fig. 1b,c). Five days after infection, the WA1-infected and Delta-infected mice were hunched or lethargic, but the Omicron-infected mice appeared completely normal (Extended Data Fig. 1a). All of the Omicron-infected mice survived the 1-week experiment; yet, 100% of the WA1-infected and 60% of the Delta-infected animals reached the humane end point during this time (Fig. 1d). This replicates previous findings from infected individuals, mice and hamsters that have shown mild disease with Omicron infection, but not with Delta and WA1 infections^1,2,20–24^.

**Fig. 2:**
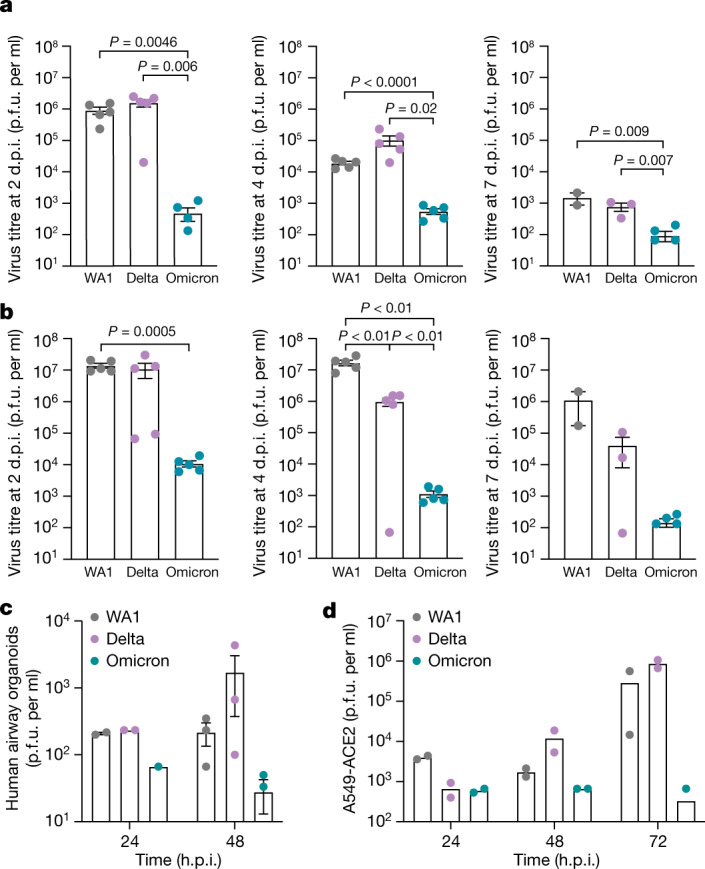
Robust viral replication of WA1 and Delta, but not Omicron, in airway cells from mice and humans. **a**, Plaque assay titres from the upper airway (nasal turbinates and bronchus) of WA1-infected, Delta-infected and Omicron-infected mice at the indicated time points. Data are shown as the average ± s.e.m. analysed by the two-tailed unpaired Student’s *t*-test. Each dot represents an infectious virus titre in an individual mouse at 2 d.p.i. (*n* = 5), 4 d.p.i. (*n* = 5) and 7 d.p.i. (WA1 infection group *n* = 2, Delta *n* = 2 and Omicron *n* = 5). **b**, Plaque assay titres from the lungs of infected mice at the indicated time points. Data are shown as the average ± s.e.m. at each time point and were analysed by the two-tailed unpaired Student’s *t*-test. Each dot represents infectious virus titre in individual mice at 2 d.p.i. (*n* = 5), 4 d.p.i. (*n* = 5) and 7 d.p.i. (WA1 infection group *n* = 2, Delta *n* = 2 and Omicron *n* = 5). **c**, Plaque assay titres from supernatants of infected human airway organoids (multiplicity of infection (MOI) of 1). Data are shown as the average ± s.e.m. Each dot represents an independent experiment using human lung airway organoids generated at 24 h (*n* = 2) and 48 h (*n* = 3). h.p.i., hours post-infection. **d**, Plaque assay titres from supernatants of infected A549-ACE2 cells (MOI of 0.1). *n* = 2 represents infectious virus titres in independent experiments. Source Data

**Extended Data Fig. 2:**
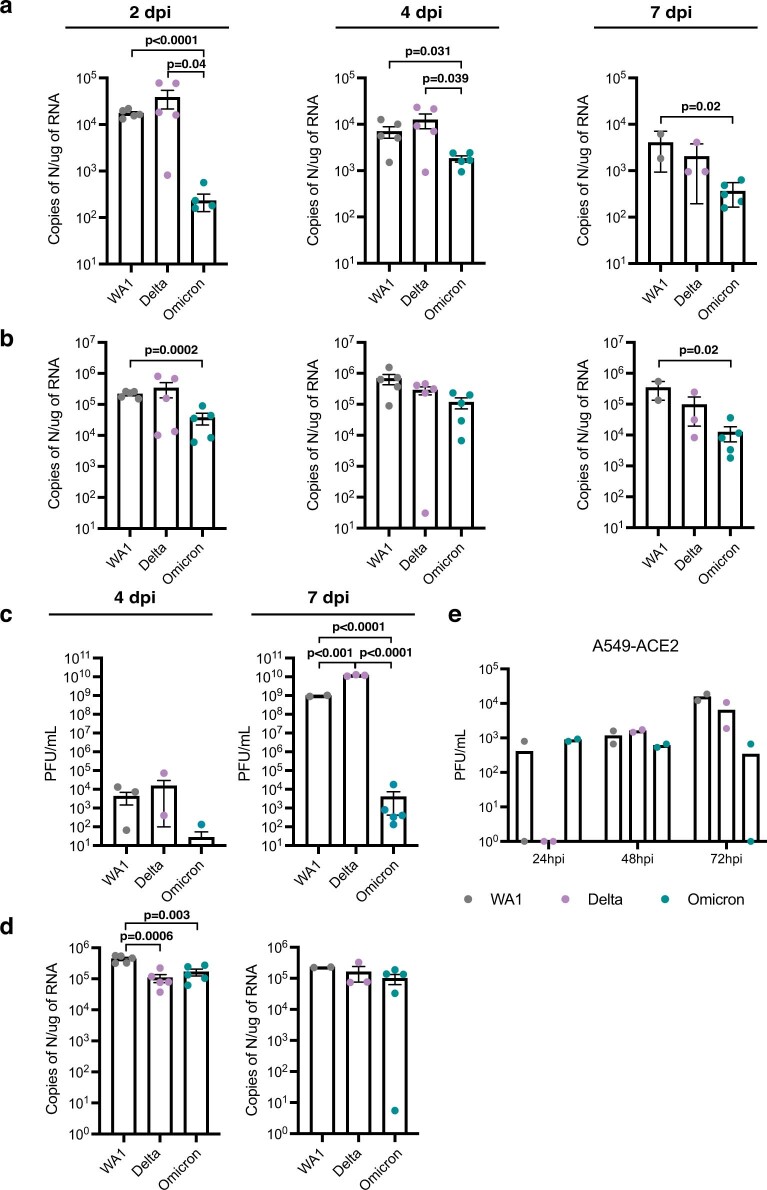
Lower viral replication of Omicron in mice and human cells. **a**, RT-qPCR of SARS-CoV-2 N RNA isolated from upper respiratory tract (nasal turbinates and bronchus) of WA1-(grey), Delta-(purple), and Omicron-(teal) infected mice at indicated time points. Data are expressed in absolute copies/μg based on a standard curve of N gene with known copy number. Data are shown as an average ± SEM at 2 dpi (n = 5), 4 dpi (n = 5), and 7 dpi (WA1 infection group n = 2, Delta n = 2 and Omicron n = 5) and analyzed by two-tailed unpaired student’s t-test. **b**, RT-qPCR of SARS-CoV-2 N RNA isolated from lungs of infected mice at indicated time points. Data are expressed in absolute copies/μg based on a standard curve of N gene with known copy number. Data are shown as an average ± SEM at 2 dpi (n = 5), 4 dpi (n = 5), and 7 dpi (WA1 infection group n = 2, Delta n = 2 and Omicron n = 5) and analyzed by the two-tailed unpaired student's t-test. **c**, Plaque assay titers from the brains of infected mice at indicated time points. Data are shown as an average ± SEM at 4 dpi (n = 5) and 7 dpi (WA1 infection group n = 2, Delta n = 2 and Omicron n = 5) and analyzed by the two-tailed unpaired student’s t-test. **d**, RT-qPCR of SARS-CoV-2 N RNA isolated from brains of infected mice at indicated time points. Data are expressed in absolute copies/μg based on a standard curve of N gene with known copy number. Data are shown as an average ± SEM at 4 (n = 5) and 7 (n = 2–5) dpi and analyzed by the two-tailed unpaired student's t-test. **e**, Plaque assay titers from supernatants of infected A549-ACE2 (MOI of 0.01). Data are shown as average ± SEM, n = 2. Source Data

To assess viral replication dynamics, we quantified infectious particle production (Fig. 2a,b) and viral RNA expression (Extended Data Fig. 2a,b) in the respiratory tracts and lungs of infected mice over time. Across all time points, high titres of infectious virus were present in the upper airways (nasal turbinates and bronchi) and lungs of WA1-infected and Delta-infected mice, whereas Omicron replication was significantly lower in these organs, as previously reported^20–22^. Lung histology showed that Omicron infection resulted in small localized foci of infected cells (marked by nucleocapsid staining (green)) (Extended Data Fig. 1b–d). A similar pattern but with enhanced numbers were observed after WA1 infection, and Delta infection showed large patches of infected cells, indicative of enhanced cell-to-cell spread, as reported in human lung organoids and cell lines^11^ (Extended Data Fig. 1b–d). In addition, brain tissue, which is a target for viral replication in K18-hACE2 mice, showed lower Omicron replication 4 and 7 days after infection. Omicron infection also produced fewer infectious particles in human airway organoids and the human alveolar A549 epithelial cell line overexpressing ACE2 than WA1 and Delta infections (Fig. 2c,d), which is consistent with our findings in mice.

## Immune markers differ between variants

**Extended Data Fig. 3:**
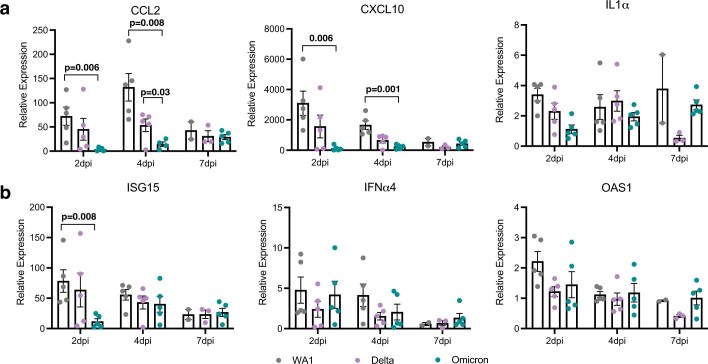
Differential expression of proinflammatory markers in lungs of infected mice. **a**, RT-qPCR of proinflammatory cytokines and chemokines from RNA isolated from lungs of infected mice at the indicated time points. Data are expressed relative to mock-infected mice. Data are shown as the average ± SEM at 2 dpi (n = 5), 4 dpi (n = 5), and 7 dpi (WA1 infection group n = 2, Delta n = 2 and Omicron n = 5) and analyzed by two-tailed unpaired student’s t-test. **b**, RT-qPCR of interferon-stimulated genes from RNA isolated from lungs of infected mice at the indicated time points. Data are expressed relative to mock-infected mice. Data are shown as the average ± SEM at 2 dpi (n = 5), 4 dpi (n = 5), and 7 dpi (WA1 infection group n = 2, Delta n = 2 and Omicron n = 5) and analyzed by the two-tailed unpaired student’s t-test. Source Data

As severe COVID-19 is associated with cytokine storms in conjunction with exhaustion of T cells^25^, we next assessed cytokine expression and T cell phenotypes in infected mouse lungs. Although infection with WA1 and Delta readily induced pro-inflammatory markers of severe COVID, such as CXCL10 and CCL2 (ref. ^26^), induction by Omicron was significantly reduced early after infection (Extended Data Fig. 3a). Induction of IL-1α was not significantly different between the three viral isolates, but trended towards lower expression in Omicron-infected animals 2 days post-infection (Extended Data Fig. 3a). Although no significant differences between the viral variants were observed in the induction of IFNα or relevant downstream induced genes, such as interferon-stimulated gene 15 (*ISG15*) and 2'-5'-oligoadenylate synthetase 1 (*OAS1*), we cannot exclude that this is caused by a low number of animals at later time points (Extended Data Fig. 3a,b).

**Fig. 3:**
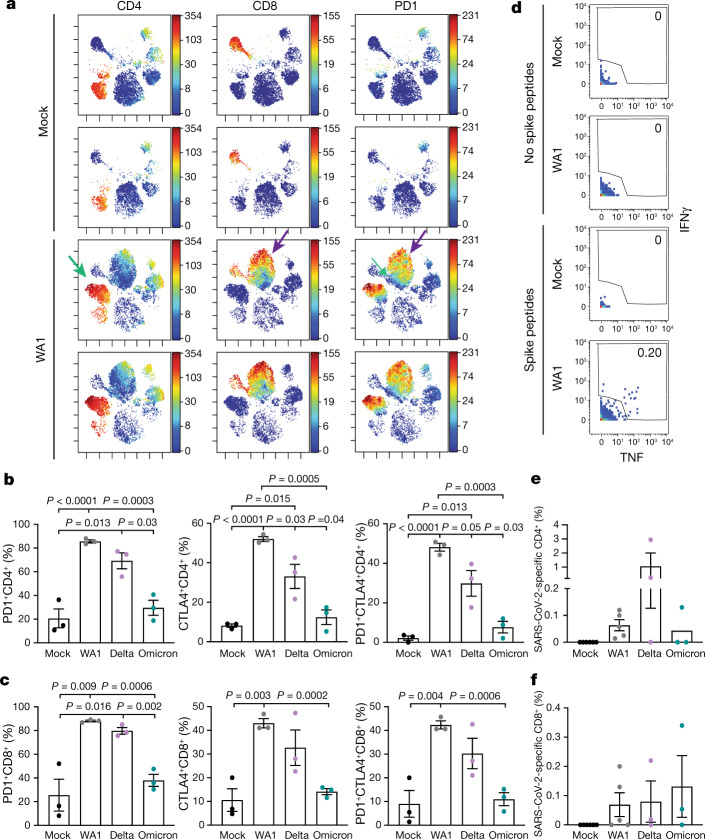
Ancestral and VOC SARS-CoV-2 elicit immune responses in the lungs of mice. **a**, T cells from lungs of infected mice (*n* = 3) were phenotypically distinct and expressed PD1. Single-cell suspensions of lungs from mock-infected and WA1-infected K18-hACE2 mice were harvested at 9 d.p.i. and analysed by CyTOF. Shown are tSNE plots gated on total immune cells (CD45^+^) from the lungs of mice, coloured by expression levels of the antigen listed at the top (red shows the highest expression and blue represents the lowest expression). 'Islands' of CD4^+^ and CD8^+^ T cells unique to the infected mice (identified by green and purple arrows, respectively, in the third row) express especially high levels of the activation/exhaustion marker PD1, as demonstrated in the right-hand column. **b**,**c**, T cells from lungs of infected mice (*n* = 3) expressed elevated levels of the activation/checkpoint antigens PD1 and CTLA4. The proportions of CD4^+^ (**b**) and CD8^+^ (**c**) T cells expressing PD1, CTLA4 or both PD1 and CTLA4 are indicated. **d**, SARS-CoV-2-specific T cells are elicited in the lungs of mice infected with SARS-CoV-2. Representative plots corresponding to pulmonary T cells from uninfected (mock) and WA1-infected K18-hACE2 mice, stimulated for 6 h with or without overlapping SARS-CoV-2 spike peptides. Note that SARS-CoV-2-specific T cells (those producing IFNγ and/or TNF) were only detected in infected mice after peptide stimulation (*n* = 3). **e**,**f**, SARS-CoV-2-specific T cells are elicited in the lungs of mice infected with WA1 (*n* = 6), Delta (*n* = 3) and Omicron (*n* = 3). The proportions of CD4^+^ (**e**) and CD8^+^ (**f**) T cells expressing IFNγ and/or TNF (gated as shown in **c**) are indicated. CD4^+^ T cell responses trended highest in Delta-infected mice, and the CD8^+^ T cell responses were highest in Delta-infected and Omicron-infected mice (*n* = 3). In **b**,**c**,**e**,**f**, data are shown as the average ± s.e.m. analysed by the two-tailed unpaired Student’s *t*-test. Source Data

To determine whether the pro-inflammatory response that we observed in WA1-infected mice is also associated with T cell exhaustion in late infection, we generated single-cell suspensions from the lungs of mock-infected and WA1-infected mice, and performed cytometry by time of flight (CyTOF) mass spectrometry before and after stimulation with overlapping 15-mer peptides that span the entire spike protein. tSNE visualization of the CyTOF data corresponding to total immune (CD45^+^) cells from the unstimulated specimens revealed that both CD4^+^ and CD8^+^ T cells of infected mice segregate distinctly from their respective counterparts in the mock-infected mice, indicating profound phenotypic changes in pulmonary T cells upon WA1 infection, including upregulation of the activation/exhaustion marker programmed cell death 1 (PD1) on T cells from the infected animals (Fig. 3a).

When similar experiments were performed with infections by WA1, Delta and Omicron, we found elevated expression levels of not only PD1 but also cytotoxic T lymphocyte-associated protein 4 (CTLA4; which is another activation/exhaustion marker) on pulmonary T cells in all infected animals, although to a significantly lesser extent in the Omicron-infected mice (Fig. 3b,c). Despite evidence of pulmonary T cell exhaustion in mice infected with all three isolates, functional SARS-CoV-2-specific T cells were still generated in the lungs, as demonstrated by our identification of IFNγ-producing and TNF-producing cells specifically in the peptide-stimulated specimens (Fig. 3d–f). These results suggest that the diminished pro-inflammatory cytokines and activated/exhausted pulmonary T cells elicited by Omicron associate with diminished Omicron pathogenicity and the 2–3 logs decrease in Omicron replication.

## Cross-variant neutralization

**Fig. 4:**
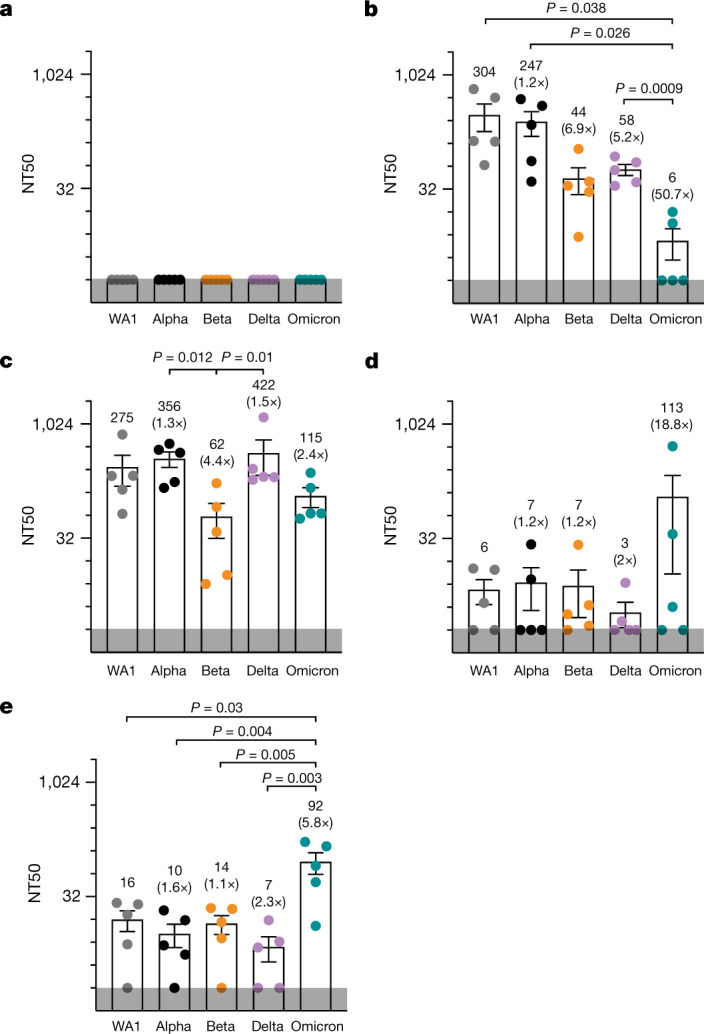
Cross-variant neutralization of SARS-CoV-2 isolates from the sera of infected mice. K18-hACE2 mice were infected with 1 × 10^4^ p.f.u. of WA1, Delta or Omicron. The virus neutralization assay was carried out with sera collected at 7 d.p.i. Data points in the graph represent individual sera samples showing NT50s against SARS-CoV-2 isolates. The numbers in parentheses indicate the fold change in neutralization efficacy or resistance of respective isolates relative to the NT50 of the ancestral isolate (WA1). The grey band at the bottom of the graph indicates the limit of detection. **a**–**d**, Graphs representing the NT50 of sera from naive (**a**), WA1-infected (**b**), Delta-infected (**c**) and Omicron-infected (**d**) mice against different viral isolates. *n* = 5 mouse in each group. **e**, K18-hACE2 mice were infected with 5 × 10^2^ p.f.u. of Omicron (*n* = 5). The virus neutralization assay was carried out with sera collected at 9 d.p.i. Data are presented as average ± s.e.m. and were analysed by two-way ANOVA and two-tailed unpaired Student’s *t*-test. Source Data

To determine humoral immune responses induced by infection with the three different isolates, we collected sera from mice at 7 days after infection and tested their neutralization efficiency against SARS-CoV-2 isolates: WA1, Alpha, Beta, Delta and Omicron. We determined the p.f.u. at different serum dilutions and calculated the 50% neutralization titres (NT50s) (Fig. 4 and Extended Data Fig. 5). As expected, sera from uninfected mice showed no neutralization across all variants (Fig. 4a). Sera from WA1-infected mice showed effective neutralization of WA1 and Alpha and, to a lesser extent, Beta and Delta isolates, but no efficacy against Omicron (NT50 of 6) (Fig. 4b). By contrast, sera from Delta-infected mice effectively neutralized Delta (NT50 of 422), WA-1 (NT50 of 275), Alpha (NT50 of 356) and, to a lesser extent, Omicron (NT50 of 115) and Beta (NT50 of 62), with the latter significantly decreased, compared to Delta and Alpha (Fig. 4c). Omicron infection, however, only induced neutralization of Omicron (NT50 of 113), but no other isolate (NT50 of 3–7) (Fig. 4d). This was repeated and confirmed in a second experiment in which 9 days after infection, all mice infected with Omicron showed significant serum neutralization of Omicron (NT50 of 92), but no other VOC (NT50 of 7–16) (Fig. 4e). These results indicate limited cross-variant neutralization induced by Omicron relative to other isolates, which may be due to its highly mutated spike protein or its lower replicative capacity (Fig. 2). Delta and WA1, despite having similar replicative and inflammatory capacities, exhibited different neutralization profiles, underscoring the role of the different spike (and possibly other viral) proteins in eliciting cross-variant neutralization.

**Fig. 5:**
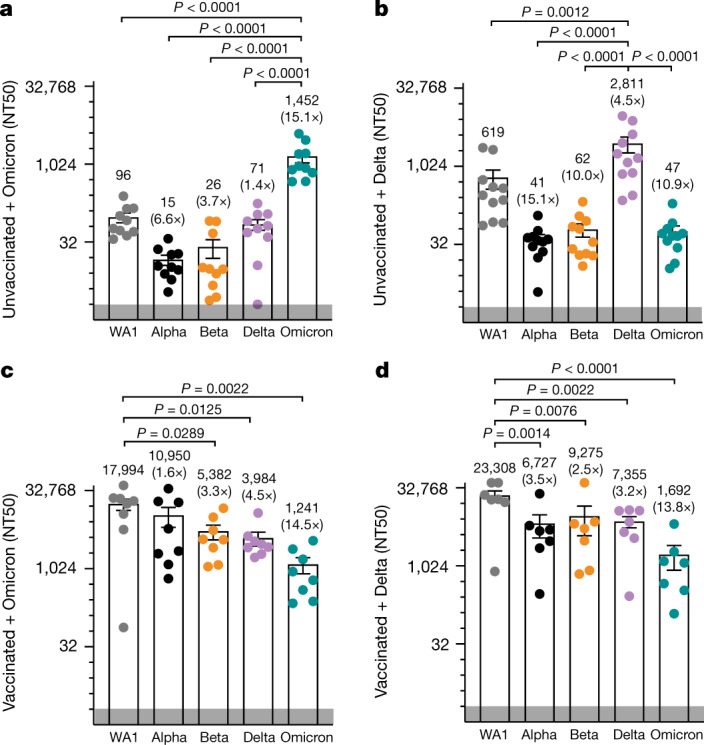
Cross-variant neutralization of SARS-CoV-2 isolates from human sera. **a**–**d**, Graphs representing the NT50 of variants by sera from unvaccinated individuals with likely Omicron infection (based on date of collection; *n* = 10) (**a**), unvaccinated individuals with likely Delta infection (based on date of collection; *n* = 11) (**b**), vaccinated individuals with a confirmed Omicron infection (*n* = 8) (**c**) and vaccinated individuals with confirmed Delta infection (based on date of collection; *n* = 7) (**d**). The data points in the graph represent individual serum samples. The grey band at the bottom of the graph indicates the limit of detection. Data presented in a–d are average ± s.e.m. and were analysed by two-way ANOVA and two-tailed unpaired Student’s *t*-test. The details regarding samples (group, age, sex, COVID-19 infection status, vaccination dates, and sample collection dates after infection or symptoms are summarized in Extended Data Table 1). Source Data

**Extended Data Fig. 4:**
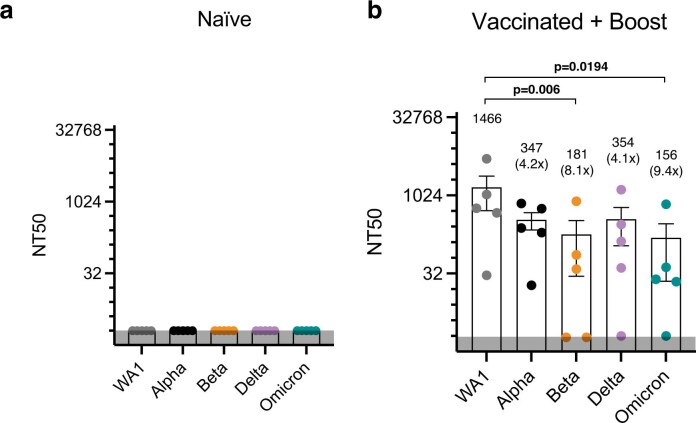
Cross-variant neutralization of SARS-CoV-2 isolates by human sera. Graphs representing NT50 of sera from **a**, naive and **b**, vaccinated and Pfizer-boosted individuals against different viral isolates, n = 5 in each group. The average neutralization efficacies of sera from each graph are shown and fold-changes relative to ancestral isolate (WA1) are shown in parentheses. The grey band indicates the limit of detection. Data are shown as the average ± SEM and analyzed by 2-way ANOVA and adjusted for multiple testing using the Bonferroni test. Source Data

**Extended Data Fig. 5:**
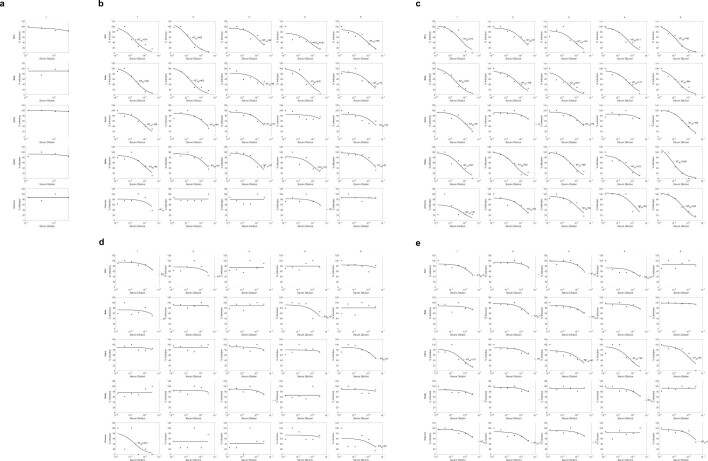
Sera neutralizing titer assays of from SARS-CoV-2-infected mice. Neutralization assays of sera from **a**, naive (representative), **b**, WA1-, **c**, Delta-, and **d**, Omicron-infected mice at 7 days post-infection against WA1, Alpha, Beta, Delta, and Omicron isolates. **e**, Neutralization assays of sera from Omicron-infected mice at 9 days post-infection against WA1, Alpha, Beta, Delta, and Omicron isolates.

**Extended Data Fig. 6:**
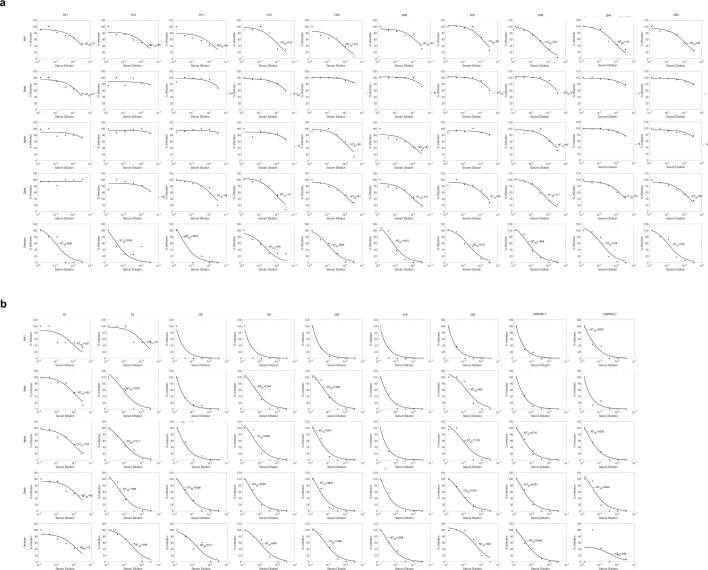
Sera neutralizing titer assays of individuals infected with Omicron. Neutralization assays of sera from a, unvaccinated and b, vaccinated individuals infected with Omicron (likely based on time of collection) against WA1, Alpha, Beta, Delta, and Omicron isolates.

**Extended Data Fig. 7:**
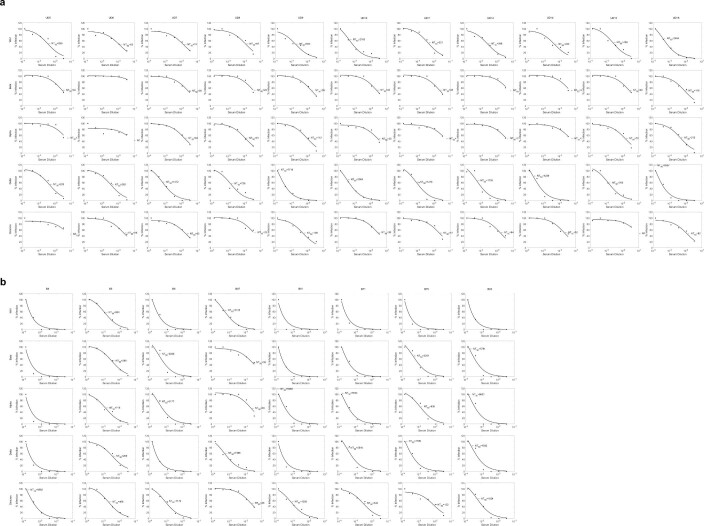
Sera neutralizing titer assays from individuals infected with Delta. Neutralization assays of sera from **a**, unvaccinated and **b**, vaccinated individuals infected with Delta (likely based on time of collection) against WA1, Alpha, Beta, Delta, and Omicron isolates.

These data were confirmed with sera from 10 unvaccinated individuals who had recovered from Omicron infection (Extended Data Table 1). These sera showed the same limited cross-variant neutralization as observed in mice with effective neutralization of only Omicron itself (NT50 of 1,452) and an approximately 15-fold decrease in neutralizing titres against non-Omicron isolates (NT50 of 15–96) (Fig. 5a and Extended Data Fig. 6). Analysis of sera from 11 matched, unvaccinated individuals with Delta infection showed a similar pattern: highest neutralization of Delta itself (NT50 of 2,811), followed by WA1 (NT50 of 619) and decreased neutralization of Alpha, Beta and Omicron (NT50 of 41–62) (Fig. 5b and Extended Data Fig. 7). Sera from uninfected, unvaccinated individuals showed no neutralization across all variants as expected (Extended Data Fig. 4a).

**Extended Data Fig. 8:**
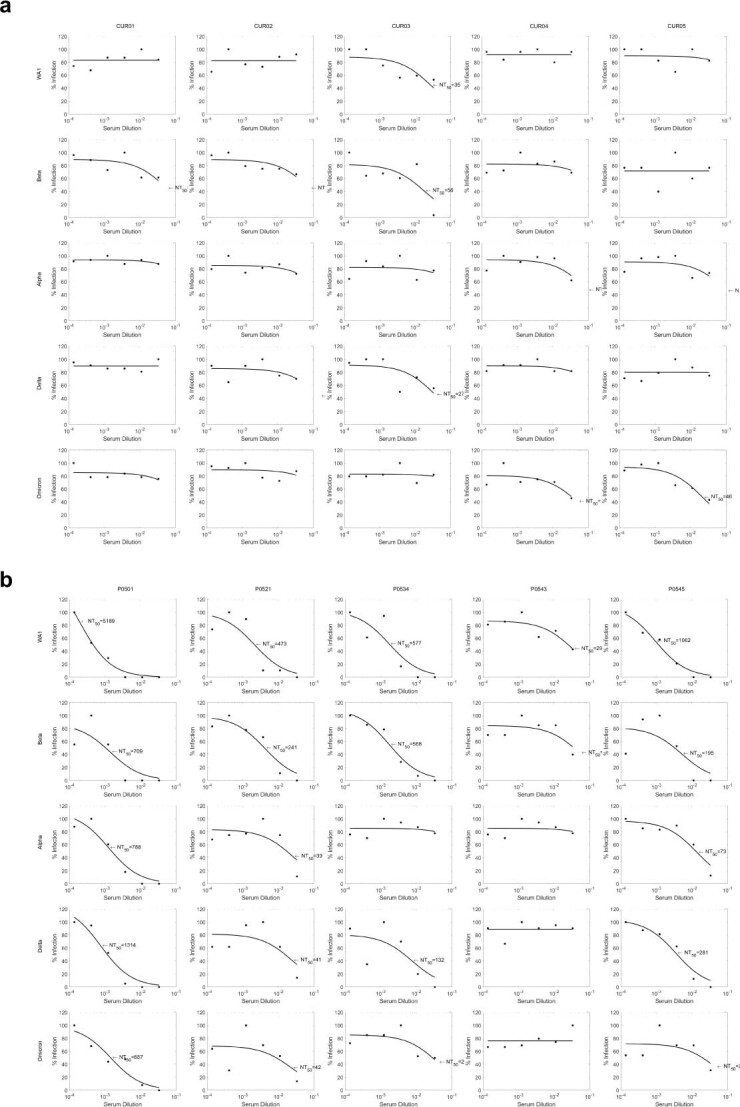
Sera neutralizing titer assays from individuals. Neutralization assays of sera from **a**, naive and **b**, vaccinated and Pfizer boosted individuals against WA1, Alpha, Beta, Delta, and Omicron isolates.

Sera from vaccinated individuals with confirmed Omicron or Delta breakthrough infections showed high neutralizing titres against all isolates, with highest titres against WA1 (NT50 of 17,994 and 23,308) and lowest against Omicron (NT50 of 1,241 and 1,692) (Fig. 5c,d). These values exceeded neutralizing titres induced by the third shot of Pfizer/BioNTech vaccines, in which titres were, on average, 10 times lower than those observed after breakthrough infections (Extended Data Figs. 4b and 8). These results suggest that Omicron and Delta breakthrough infections can boost existing immunity conferred by vaccination, thereby eliciting a form of 'hybrid immunity' that is effective against not only itself but also other SARS-CoV-2 variants.

Collectively, our study shows that, although the Omicron variant is immunogenic, infection in unvaccinated individuals may not elicit effective cross-neutralizing antibodies to non-Omicron variants. However, in vaccinated individuals, Omicron infection effectively induces immunity against itself and enhances neutralization of other variants. This, together with our finding that Delta infection also elicits broad cross-variant neutralization in vaccinated individuals, supports the inclusion of Omicron-based and Delta-based immunogens in future heterologous or multivalent vaccination strategies for broad protection against variants.

## Methods

### Human lung organoids

Whole human lung tissue was digested to a single-cell suspension and plated in basement membrane extract as previously published^27^. In brief, organoids were maintained in DMEM supplemented with supplemented with 10% (vol/vol) R-spondin1 conditioned medium, 1% B27 (Gibco), 25 ng ml^−1^ noggin (Peprotech), 1.25 mM *N*-acetylcysteine (Sigma-Aldrich), 10 mM nicotinamide (Sigma-Aldrich), 5 nM herefulin-β1 (Peprotech) and 100 µg ml^−1^ Primocin (InvivoGen). HAO medium was further supplemented with 5 µM Y-27632, 500 nM A83-01, 500 nM SB202190, 25 ng ml^−1^ FGF7 and 100 ng ml^−1^ FGF10 (all from Stem Cell Technologies). HAO medium was replaced every 3–4 days.

A549 cells expressing ACE2 (A549-ACE2) from the American Type Culture Collection (ATCC) and Vero cells expressing TMPRSS (Vero-TMPRSS2) were a gift from O. Schwartz and S. P. J. Whelan, respectively. A549-ACE2 and Vero-TMPRSS2 cells were cultured in DMEM supplemented with 10% FBS and blasticidin (20 μg ml^−1^) (Sigma) at 37 °C and 5% CO_2_. Short terminal repeat analysis by the Berkeley Cell Culture Facility authenticated these as A549 cells with 100% probability. The cells are free of mycoplasma contamination.

Vero cells stably co-expressing human ACE2 and TMPRSS2 (a gift from A. Creanga and B. Graham at the US National Institutes of Health) were maintained at 37 °C and 5% CO_2_ in DMEM (Gibco) supplemented with 10% fetal calf serum, 100 μg ml^−1^ penicillin and streptomycin (Gibco) and 10 μg ml^−1^ puromycin (Gibco). The cells are free of mycoplasma contamination.

### SARS-CoV-2 virus culture

SARS-CoV-2/human/USA/USA-WA1/2020 (WA1) (BEI NR-52281), B.1.1.7 (California Department of Health), B.1.351 (BEI NR-54008), B.1.617.2 (California Department of Health) and B.1.1.529 (California Department of Health; BA.1) were used for animal infection studies or serum virus neutralization. The virus infection experiments were performed in a biosafety level 3 laboratory. Working stocks of SARS-CoV-2 were made in Vero-TMPRSS2 cells and were stored at −80 °C until used.

The Omicron variant was isolated from a nasopharyngeal swab sample from a patient hospitalized with COVID-19 at the University of California, San Francisco (UCSF). A 200 μl aliquot of the sample was serially diluted 1:1 with medium (DMEM supplemented with 1× penicillin–streptomycin) in a 96-well plate for five dilutions, in duplicate. Then, 100 μl of freshly trypsinized Vero-hACE2–TMPRSS2 cells, resuspended in infection medium (made as above but with 2× penicillin–streptomycin, 5 μg ml^−1^ amphotericin B (Bioworld) and no puromycin) were added to the nasal sample dilutions at a concentration of 2.5 × 10^5^ cells per ml. Cells were cultured at 37 °C and 5% CO_2_ and checked for cytopathic effects (CPEs) from days 2 to 3. Vero-hACE2–TMPRSS2 cells formed characteristic syncytia upon infection with SARS-CoV-2, enabling rapid and specific visual evaluation for CPEs. Supernatants were harvested on day 3 after inoculation. A 200 μl aliquot of passage 0 (P0) was used to infect a confluent T25 flask to generate a P1 culture, harvested after 3 days. Virus stocks were titred by plaque assay, and the sequence was confirmed by nanopore sequencing.

### K18-hACE2 mouse infection model

All protocols concerning animal use were approved (AN169239-01C) by the Institutional Animal Care and Use committees at the University of California, San Francisco and Gladstone Institutes and conducted in strict accordance with the National Institutes of Health Guide for the Care and Use of Laboratory Animal^28^. Mice were housed in a temperature-controlled (65–75 °F) and humidity-controlled (40–60%) pathogen-free facility with a 12-h light–dark cycle and ad libitum access to water and standard laboratory rodent chow.

In brief, the study involved intranasal infection (1 × 10^4^) of 6–8-week-old female K18-hACE2 mice with Delta and Omicron, and WA1 served as a control isolate of SARS-CoV-2. A total of 15 animals were infected for each variant. Five mice from each group were euthanized at 2, 4 and 7 days post-infection. The brain, lungs and upper respiratory tract, including bronchus and nasal turbinates, were processed for further analysis of virus replication.

### Cellular infection studies

A549-ACE2 cells were seeded into 12-well plates. Cells were rested for at least 24 h before infection. At the time of infection, medium containing viral inoculum (MOI of 0.01 and 0.1) was added on the cells. At 1 h after addition of inoculum, the medium was replaced with fresh medium without viral inoculum. Supernatants were harvested at 24, 48 and 72 h post-infection for further plaque assays.

### Organoid infection studies

Organoids were plated on geltrex-coated plates (12760013, Thermo Fisher) with 100,000 cells per well, and infected at an MOI of 1. At 2 h after addition of the inoculum, the supernatant was removed, cells were washed with PBS and fresh HAO medium was added. Supernatants were harvested for a plaque assay at 24 and 48 h.

### Virus neutralization assay

K18-hACE2 mice were infected with 1 × 10^4^ p.f.u. of WA1, B.1.617.2 and B.1.1.529 (*n* = 5). With the early humane end points with WA1 and B.1.617.2, more animals (*n* = 15) were infected for these groups. Serum samples from mice were collected at 7 days post-infection. Mock-infected animals served as controls. Serum dilutions (50 µl) were made to get final dilutions of 1:30, 1:90, 1:270, 1:810, 1:2,430 and 1:7,290 in serum-free DMEM. Dilutions were separately added with 50 p.f.u. (50 µl) of SARS-CoV-2 WA1, Alpha, Beta, Delta and Omicron. The mixture was mixed gently, incubated at 37 °C for 30 mins, followed by a plaque assay. Similar assays were performed for serum samples from Omicron-infected (5 × 10^2^) mice obtained at 9 d.p.i., and human serum samples acquired from ongoing clinical trials led by Curative and the UCSF or from hospitalized patients at the UCSF (Extended Data Table 1). The virus neutralization efficacy of sera was presented as NT50 and the average of each variant, and compared to others in terms of fold change. NT50 graphs were generated by MATLAB (version 9.12). Data analysis was performed by using GraphPad Prism version 9.3.

### Plaque assays

Tissue homogenates and cell supernatants were analysed for viral particle formation for in vivo and in vitro experiments, respectively. In brief, Vero-TMPRSS2 cells were seeded and incubated overnight. Cells were inoculated with 10^−1^ to 10^−6^ dilutions of the respective homogenates or supernatant in serum-free DMEM. After the 1-h absorption period, the media in the wells were overlaid with 2.5% Avicel (RC-591, Dupont). After 72 h, the overlay was removed, and the cells were fixed in 10% formalin for 1 h and stained with crystal violet for visualization of p.f.u. Data analysis was performed by using GraphPad Prism version 9.3.

### Quantitative PCR

RNA was extracted from cells, supernatants or tissue homogenates with RNA-STAT-60 (CS-110, AMSBIO) and the Direct-Zol RNA Miniprep Kit (R2052, Zymo Research). RNA was then reverse-transcribed to cDNA with iScript cDNA Synthesis Kit (1708890, Bio-Rad). Quantitative PCR was performed with cDNA and SYBR Green Master Mix (Thermo Scientific) using the CFX384 Touch Real-Time PCR Detection System (Bio-Rad). See Extended Data Table 2 for primers sequences. N gene standards were used to generate a standard curve for copy number quantification. N gene standard was generated by PCR using extracted genomic SARS-CoV-2 RNA as template. A single product was confirmed by gel electrophoresis, and DNA was quantified by Nanodrop.

### CyTOF analysis of mouse lung specimens

The mice used in the CyTOF study were infected with 5 × 10^2^ p.f.u. of WA1 and monitored for clinical signs of infection (for example, body weight and body temperature) starting from 1 to 9 days post-infection. CyTOF was conducted as described^29^. Single-cell suspensions of lung tissue specimens processed using the GentleMACS system (Miltenyi) were treated with 25 μM cisplatin (Sigma) for 60 s as a viability dye. Cells were then quenched with CyFACS buffer (PBS supplemented with 0.1% BSA and 0.1% sodium azide) and fixed for 10 min with 2% paraformaldehyde (PFA; Electron Microscopy Sciences). Cells were then washed twice with CyFACS buffer (PBS with 0.1% BSA, 0.05% sodium azide, and 2 mM EDTA) and frozen at −80 °C until CyTOF antibody staining. Before antibody staining, specimens were barcoded using the Cell‐ID 20‐Plex PD Barcoding kit (Fluidigm). Fc blocking was performed by treating the cells with 1.5% mouse and rat sera (both from Thermo Fisher) for 15 min at 4 °C. After washing with CyFACS, cells were stained for 45 min at 4 °C with the cell-surface antibodies listed in Extended Data Table 3. Antibodies were purchased pre-conjugated from Fluidigm or conjugated using the Maxpar X8 antibody labelling kit (Fluidigm). After staining, cells were washed with CyFACS and fixed overnight at 4 °C in 2% PFA and permeabilized for 30 min with Foxp3 fix/permeabilization buffer (Fisher Scientific). After two washes with permeabilization buffer (Fisher Scientific), cells were Fc blocked again for 15 min at 4 °C with mouse and rat sera diluted in permeabilization buffer. After washing with permeabilization buffer, cells were stained for 45 min at 4 °C with the intracellular antibodies listed in Extended Data Table 3. The details about the antibody dilutions have been provided in the Extended Data Table 3. Before CyTOF analysis, cells were incubated for 20 min with a 1:500 dilution DNA intercalator (Fluidigm), and then washed twice with CyFACS and once with Cell Acquisition Solution (CAS; Fluidigm). Acquisition was performed in the presence of EQ Four Element Calibration Beads (Fluidigm) diluted in CAS. Cells were analysed on a CyTOF 2 instrument (Fluidigm) at the UCSF Parnassus Flow Core. For data analysis, CyTOF datasets were normalized to EQ calibration using CyTOF software (6.7.1014, Fluidigm) and manually gated using the FlowJo software (10.7.2, FlowJo LLC, BD Biosciences). tSNE visualizations of the datasets were performed in Cytobank (9.1, 2022 Cytobank, Inc.), with default settings.

### Histology

Mouse lung tissues were fixed in 4% PFA (47608, Sigma) for 24 h, washed three times with PBS and stored in 70% ethanol. In brief, tissues were processed and embedded in paraffin, and tissue sections were stained for SARS-CoV-2 nucleocapsid (GTX135357, Genetex). The sections were then imaged using Leica Aperio ImageScope.

### Human serum samples

Human serum samples were acquired from two ongoing clinical trials led by Curative and the UCSF. The Curative clinical trial protocol was approved by Advarra under Pro00054108 for a study designed to investigate immune escape by SARS-CoV-2 variant (University of California, Los Angeles protocol record PTL-2021-0007; ClinicalTrials.gov identifier NCT05171803). Sample specimens were collected from adults (18–50 years of age) who either had been vaccinated for COVID-19 and/or had a history of COVID-19. Sample acquisition involved the standard venipuncture procedure to collect a maximum of 15 ml of whole blood, incubation at ambient temperature for 30–60 min to coagulate, centrifugation at 2,200–2,500 rpm for 15 min at room temperature, and storage on ice until delivered to the laboratory for serum aliquoting and storage at −80 °C until use. A quantitative SARS-CoV-2 IgG ELISA was performed on serum specimens (anti-SARS-CoV-2 ELISA (IgG), 2606–9621G, New Jersey, EuroImmun). Remnant plasma samples from patients hospitalized with COVID-19 at the UCSF were obtained from UCSF clinical laboratories daily, based on availability. Remnant samples were aliquoted and biobanked and the retrospective medical chart review for relevant demographic and clinical metadata were performed under a waiver of consent and according to 'no subject contact' protocols approved by the UCSF Institutional Review Board (protocol number 10-01116). Plasma samples were also collected through the UMPIRE (UCSF employee and community member immune response) study (protocol number 20-33083), a longitudinal COVID-19 research study focused on collection of prospective whole-blood and plasma samples from enrolled participants to evaluate the immune response to vaccination, with and without boosting, and/or vaccine breakthrough infection. The study cohorts included fully vaccinated individuals with either two doses of emergency use authorization-authorized mRNA vaccine (Pfizer or Moderna). Consented participants came to a UCSF CTSI Clinical Research Service (CRS) laboratory where their blood was drawn by nurses and phlebotomists. At each visit, two to four 3-ml EDTA tubes of whole blood were drawn, and one or two EDTA tubes were processed to plasma from each time point. Relevant demographic and clinical metadata from UMPIRE participants were obtained through participant Qualtric surveys performed at enrolment and at each blood draw. Serum samples were heat inactivated at 56 °C for 30 min before use in neutralization assays.

For adequate sample selection, the criteria were age, disease severity and days after infection for serum collection. A Wilcoxon–Mann–Whitney significance test was performed between the unvaccinated plus Omicron-infected and unvaccinated plus Delta-infected individuals, which showed no statistical significance in serum collection days after infection (*P* = 0.147540), disease severity index (*P* = 0.820174) and age of the individuals (*P* = 0.591680). A Wilcoxon–Mann–Whitney significance test was also performed between the vaccinated plus Omicron-infected and vaccinated plus Delta-infected individuals and showed no significant difference in serum collection days after infection (*P* = 0.5267) and age of the individuals (*P* = 0.065).

### Reporting summary

Further information on research design is available in the Nature Research Reporting Summary linked to this paper.

## Online content

Any methods, additional references, Nature Research reporting summaries, source data, extended data, supplementary information, acknowledgements, peer review information; details of author contributions and competing interests; and statements of data and code availability are available at 10.1038/s41586-022-04865-0.

### Supplementary information

Reporting Summary

Source Data Figs. 1–5 and Source Data Extended Data Figs. 2–4

## Figures and Tables

**Extended Data Table 1:**
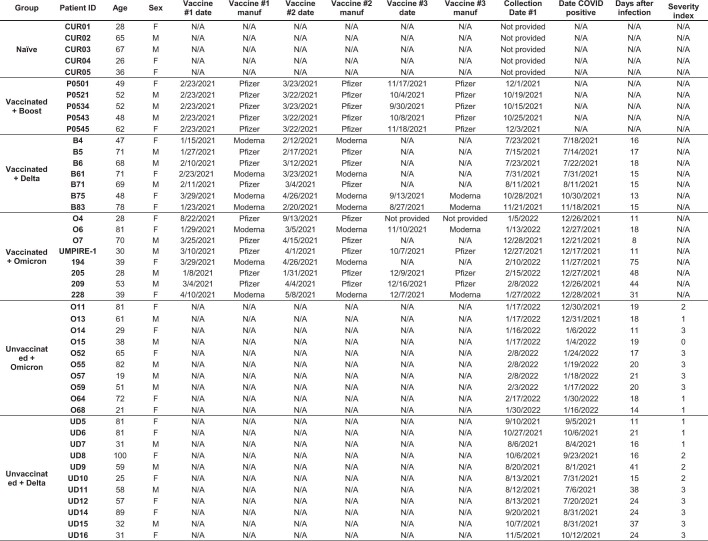
Clinical data of patients

(N = 46) included in the study. The human samples were acquired through clinical trials led by Curative Inc. and University of California, San Francisco (UCSF). F= Female, M= Male, N/A- Not applicable, severity index: 1-mild, 2-moderate, 3-severe.

**Extended Data Table 2:**
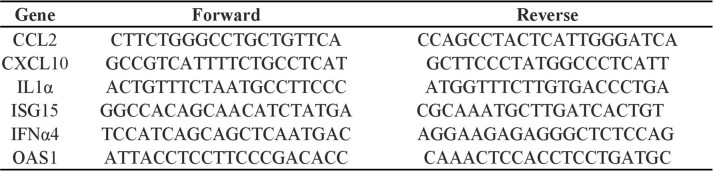
List of qPCR primers for mouse studies

The primers used to analyze cytokine expression in SARS-CoV-2- or mock-infected mice are listed.

**Extended Data Table 3:**
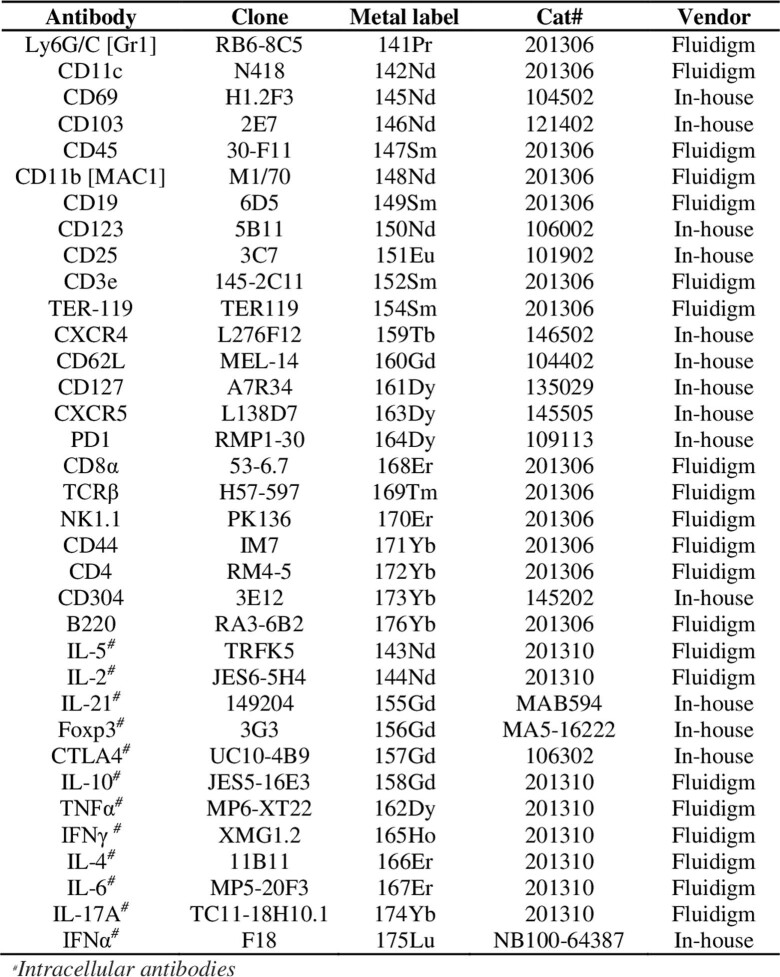
List of CyTOF-staining antibodies for mouse studies

The table describes antibody reagents used to analyze the immune response in SARS-CoV-2- or mock-infected mice by CyTOF.

## Data Availability

The datasets generated and/or analysed during the current study are available in the paper or in the Extended Data dataset.
