# Supplementary material for: Limited cross-variant immunity from SARS-CoV-2 Omicron without vaccination
Source: Nature. 2022 May 18;607(7918):351–5. doi: 10.1038/s41586-022-04865-0 (PMC9279157; doi:10.1038/s41586-022-04865-0)
Supplement: Supplementary file 1 — Reporting Summary [file 41586_2022_4865_MOESM1_ESM.pdf]

nature portfolio

Corresponding author(s): Melanie Ott, Jennifer Doudna, Charles Chiu, Nadia Roan

Last updated by author(s): Apr 29, 2022

Reporting Summary

Nature Portfolio wishes to improve the reproducibility of the work that we publish. This form provides structure for consistency and transparency in reporting. For further information on Nature Portfolio policies, see our Editorial Policies and the Editorial Policy Checklist.

Statistics

For all statistical analyses, confirm that the following items are present in the figure legend, table legend, main text, or Methods section.

n/a	Confirmed	
☐	☒	The exact sample size (n) for each experimental group/condition, given as a discrete number and unit of measurement
☐	☒	A statement on whether measurements were taken from distinct samples or whether the same sample was measured repeatedly
☐	☒	The statistical test(s) used AND whether they are one- or two-sided<br>*Only common tests should be described solely by name; describe more complex techniques in the Methods section.*
☐	☒	A description of all covariates tested
☐	☒	A description of any assumptions or corrections, such as tests of normality and adjustment for multiple comparisons
☐	☒	A full description of the statistical parameters including central tendency (e.g. means) or other basic estimates (e.g. regression coefficient) AND variation (e.g. standard deviation) or associated estimates of uncertainty (e.g. confidence intervals)
☐	☒	For null hypothesis testing, the test statistic (e.g. F, t, r) with confidence intervals, effect sizes, degrees of freedom and P value noted<br>*Give P values as exact values whenever suitable.*
☒	☐	For Bayesian analysis, information on the choice of priors and Markov chain Monte Carlo settings
☒	☐	For hierarchical and complex designs, identification of the appropriate level for tests and full reporting of outcomes
☒	☐	Estimates of effect sizes (e.g. Cohen's d, Pearson's r), indicating how they were calculated

Our web collection on statistics for biologists contains articles on many of the points above.

Software and code

Policy information about availability of computer code

Data collection	No software was used for data collection.
Data analysis	Data analysis was performed by using GraphPad Prism version 9.3. tSNE visualizations of the datasets were performed in Cytobank (9.1, 2022 Cytobank, Inc.), with default settings. CyTOF datasets were normalized to EQ calibration and manually gated using using CyTOF software (6.7.1014, Fluidigm) and manually gated using the FlowJo software (110.7.2, FlowJo LLC, BD Biosciences). NT50 graphs were generated by MATLAB (Version 9.12)

For manuscripts utilizing custom algorithms or software that are central to the research but not yet described in published literature, software must be made available to editors and reviewers. We strongly encourage code deposition in a community repository (e.g. GitHub). See the Nature Portfolio guidelines for submitting code & software for further information.

Data

Policy information about availability of data

All manuscripts must include a data availability statement. This statement should provide the following information, where applicable:

- Accession codes, unique identifiers, or web links for publicly available datasets
- A description of any restrictions on data availability
- For clinical datasets or third party data, please ensure that the statement adheres to our policy

The datasets generated during and/or analyzed during the current study are available in the manuscript or in the Extended data set.

Field-specific reporting

Please select the one below that is the best fit for your research. If you are not sure, read the appropriate sections before making your selection.

☒ Life sciences ☐ Behavioural & social sciences ☐ Ecological, evolutionary & environmental sciences

For a reference copy of the document with all sections, see nature.com/documents/nr-reporting-summary-flat.pdf

Life sciences study design

All studies must disclose on these points even when the disclosure is negative.

Sample size

For animal experiments, to estimate the minimum number of animals needed to reliably get the desired statistical significance (p<0.05) we performed power analysis and determined the animal number required for the experiment.
Experiments with human samples: The human sera experiments contained samples from a total of 46 individuals divided in six groups with n value ranged between 5-11. A Wilcoxon-Mann-Whitney significance test was performed for criteria such as age, disease severity, and days after infection for serum collection to confirm the allocation of individuals between the groups are not statistically different. We confirmed that the sample size is well powered to answer questions in this research.
Human organoid experiment: three individual experiments were performed from lung organoids generated from single human donor. The sample size was based on our previous experience of using Human organoids for SARS-CoV-2 infections.

Data exclusions

No data was excluded.

Replication

For animal experiments, we infected a group of five animals per group, per time point of the experiment and the experiment was repeated twice.
The human lung organoid experiments were performed in triplicates. All attempts during replication of experiments were successful for all the experiments except for experiments involving patients samples which were analyzed once due to limited availability

Randomization

Randomization was not relevant to this study.
For animal experiments: Animals were bred at Gladstone Institute with controlled and standardized housing and feeding conditions, Collection method of tissues or other samples was unified with covariates encoding gender and sample collection batches.
Experiments using serum from human individuals: The participants were allocated based on criteria including age, disease severity, and days after infection for serum collection. A Wilcoxon-Mann-Whitney significance test was performed to confirm the allocation of individuals between the groups do not show statistical significance.

Blinding

For human individual samples the investigators were blinded to group allocation during data collection and/or analysis.

Reporting for specific materials, systems and methods

We require information from authors about some types of materials, experimental systems and methods used in many studies. Here, indicate whether each material, system or method listed is relevant to your study. If you are not sure if a list item applies to your research, read the appropriate section before selecting a response.

Materials & experimental systems

n/a	Involved in the study	
☐	☒	Antibodies
☐	☒	Eukaryotic cell lines
☒	☐	Palaeontology and archaeology
☐	☒	Animals and other organisms
☐	☒	Human research participants
☐	☒	Clinical data
☒	☐	Dual use research of concern

Methods

n/a	Involved in the study	
☒	☐	ChIP-seq
☒	☐	Flow cytometry
☒	☐	MRI-based neuroimaging

Antibodies

Antibodies used

Antibody, Clone, Metal label, Cat# ,Vendor
Ly6G/C [Gr1] RB6-8C5 141Pr 201306 Fluidigm
CD11c N418 142Nd 201306 Fluidigm
CD69 H1.2F3 145Nd 104502 In-house
CD103 2E7 146Nd 121402 In-house
CD45 30-F11 147Sm 201306 Fluidigm
CD11b [MAC1] M1/70 148Nd 201306 Fluidigm
CD19 6D5 149Sm 201306 Fluidigm
CD123 5B11 150Nd 106002 In-house

CD25 3C7 151Eu 101902 In-house
CD3e 145-2C11 152Sm 201306 Fluidigm
TER-119 TER119 154Sm 201306 Fluidigm
CXCR4 L276F12 159Tb 146502 In-house
CD62L MEL-14 160Gd 104402 In-house
CD127 A7R34 161Dy 135029 In-house
CXCR5 L138D7 163Dy 145505 In-house
PD1 RMP1-30 164Dy 109113 In-house
CD8α 53-6.7 168Er 201306 Fluidigm
TCRβ H57-597 169Tm 201306 Fluidigm
NK1.1 PK136 170Er 201306 Fluidigm
CD44 IM7 171Yb 201306 Fluidigm
CD4 RM4-5 172Yb 201306 Fluidigm
CD304 3E12 173Yb 145202 In-house
B220 RA3-6B2 176Yb 201306 Fluidigm
IL-5# TRFK5 143Nd 201310 Fluidigm
IL-2# JES6-5H4 144Nd 201310 Fluidigm
IL-21# 149204 155Gd MAB594 In-house
Foxp3# 3G3 156Gd MA5-16222 In-house
CTLA4# UC10-4B9 157Gd 106302 In-house
IL-10# JES5-16E3 158Gd 201310 Fluidigm
TNFα# MP6-XT22 162Dy 201310 Fluidigm
IFNγ # XMG1.2 165Ho 201310 Fluidigm
IL-4# 11B11 166Er 201310 Fluidigm
IL-6# MP5-20F3 167Er 201310 Fluidigm
IL-17A# TC11-18H10.1 174Yb 201310 Fluidigm
IFNα# F18 175Lu NB100-64387 In-house

Validation

Validated by manufacture and in-house.

Eukaryotic cell lines

Policy information about cell lines

Cell line source(s)

A549-ACE2 cell line from Synthego corporation was a gift from O. Schwartz, Vero-TMPRSS2 from S.P.J. Whelan. Vero-TMPRSS2-ACE2 were gifts from A. Creanga and B. Graham at NIH. 293T-ACE2-TMPRSS2 were generated in-house using 293T cells (CRL-3216) from ATCC.

Authentication

STR analysis and western blots were performed to confirm stable expression in cell lines.

Mycoplasma contamination

Annual mycoplasma contamination tests are performed on all cell lines and all the cell line were found negative for mycoplasma contamination.

Commonly misidentified lines
(See ICLAC register)

No commonly misidentified lines were used in current study.

Animals and other organisms

Policy information about studies involving animals; ARRIVE guidelines recommended for reporting animal research

Laboratory animals

B6.Cg-Tg(K18-ACE2)2Prlmn/J animals were bred at Gladstone institute animal facility. Female mice aged between 6–8-week-old were used for SARS-CoV-2 infection studies.

Wild animals

No wild animals were used in the study.

Field-collected samples

No field collected samples were used in the study.

Ethics oversight

All animals will be housed at AAALAC accredited ABSL3 facility of Gladstone Institutes. The experimental work will be carried out in accordance with the UCSF Institutional Animal Care and Use Committee (IACUC) and the recommendations of the Panel on Euthanasia of the American Veterinary Medical Association. All protocols concerning animal use were approved (AN169239-01C) by the Institutional Animal Care and Use committees at the University of California, San Francisco and Gladstone Institutes and conducted in strict accordance with the National Institutes of Health Guide for the Care and Use of Laboratory Animal (Council, 2011). Mice were housed in a temperature (65-75°F) and humidity (40-60%) controlled pathogen-free facility with 12-hour light/dark cycle and ad libitum access to water and standard laboratory rodent chow.

Note that full information on the approval of the study protocol must also be provided in the manuscript.

Human research participants

Policy information about studies involving human research participants

Population characteristics

The human research participants (n=46) were characterized in six groups, including- Naive (n=5), Vaccinated + boost (n=5), Vaccinated + delta infected (n=7), vaccinated Omicron infected (n=8), unvaccinated + Omicron infected (n=10) and

	unvaccinated + Delta infected (n=11). All the information about the human research participants age, sex, COVID-19 infection status, vaccination dates, severity index and serum collection days after infection is provided in extended data table 1.
Recruitment	Human serum samples acquired from two ongoing clinical trials led by Curative and UCSF or from hospitalized patients at UCSF
Ethics oversight	1. Curative clinical trial protocol was approved by Advarra under Pro00054108.<br>2. UCSF Institutional Review Board approved protocol for remnant plasma samples obtained from patients hospitalized with COVID-19 at UCSF (protocol number 10-01116) and UCSF EMPloyee and community member Immune REsponse (protocol number 20-33083)

Note that full information on the approval of the study protocol must also be provided in the manuscript.

Clinical data

Policy information about clinical studies

All manuscripts should comply with the ICMJE guidelines for publication of clinical research and a completed CONSORT checklist must be included with all submissions.

Clinical trial registration	20-33083 (UCSF, protocol ID), NCT05171803 (Curative, ClinicalTrials.gov)
Study protocol	See Material and Methods.
Data collection	See Material and Methods.
Outcomes	See Material and Methods, trial ongoing.
